# Hydrographic Feature Variation Caused Pronounced Differences in Planktonic Ciliate Community in the Pacific Arctic Region in the Summer of 2016 and 2019

**DOI:** 10.3389/fmicb.2022.881048

**Published:** 2022-06-09

**Authors:** Chaofeng Wang, Mengyao Yang, Yan He, Zhiqiang Xu, Yuan Zhao, Wuchang Zhang, Tian Xiao

**Affiliations:** ^1^CAS Key Laboratory of Marine Ecology and Environmental Sciences, Institute of Oceanology, Chinese Academy of Sciences, Qingdao, China; ^2^Laboratory for Marine Ecology and Environmental Science, Qingdao National Laboratory for Marine Science and Technology, Qingdao, China; ^3^Center for Ocean Mega-Science, Chinese Academy of Sciences, Qingdao, China; ^4^College of Marine Life Sciences and Institute of Evolution and Marine Biodiversity, Ocean University of China, Qingdao, China; ^5^First Institute of Oceanography, Ministry of Natural Resources, Qingdao, China; ^6^Jiaozhou Bay Marine Ecosystem Research Station, Institute of Oceanology, Chinese Academy of Sciences, Qingdao, China

**Keywords:** planktonic ciliate, community structure, hydrographic variations, pacification, microzooplankton, Pacific Arctic Region

## Abstract

Planktonic ciliates are an important component of microzooplankton, but there is limited understanding of their responses to changing environmental conditions in the Pacific Arctic Region. We investigated the variations of ciliate community structure and their relationships with environmental features in the Pacific Arctic Region in the summer of 2016 and 2019. The Pacific water was warmer and more saline in 2019 than in 2016. The abundance and biomass of total ciliate and aloricate ciliate were significantly higher in 2019 than those in 2016, while those of tintinnid were significantly lower. The dominant aloricate ciliate changed from large size-fraction (> 30 μm) in 2016 to small size-fraction (10–20 μm) in 2019. More tintinnid species belonging to cosmopolitan genera were found in 2019 than in 2016, and the distribution of tintinnid species (*Codonellopsis frigida*, *Ptychocylis obtusa*, and *Salpingella* sp.1) in 2019 expanded by 5.9, 5.2, and 8.8 degrees further north of where they occurred in 2016. The environmental variables that best-matched tintinnid distributions were temperature and salinity, while the best match for aloricate ciliate distributions was temperature. Therefore, the temperature might play a key role in ciliate distribution. These results provide basic data on the response of the planktonic ciliate community to hydrographic variations and implicate the potential response of microzooplankton to Pacification as rapid warming progresses in the Pacific Arctic Region.

## Introduction

The Pacific Arctic Region, extending from the northern Bering Sea into the Chukchi Sea and adjacent Arctic seas, is recognized as one of the region most sensitive to global climate changes (Grebmeier and Maslowski, 2014). In recent decades, rapid changes have been found in the Arctic, such as sea ice retreat (Stroeve et al., 2012), near-surface air temperature increase (Screen and Simmonds, 2010), and increasing Pacific Inflow Water (Pacific water transport from the Bering Sea to the Arctic Ocean, including Alaskan Coastal Water, Bering Shelf Water, and Anadyr Water) (Woodgate, 2018; Lalande et al., 2021; Woodgate and Peralta-Ferriz, 2021). Pacific currents flow from the Bering Sea and transport plankton to the Arctic Ocean through the Bering Strait (Springer et al., 1996; Steele et al., 2004). A long-term increase in the annual mean transport of Pacific Inflow Water into the Arctic has been recorded in year-round, *in situ* Bering Strait mooring data, and this increase may bring more fresh water and heat fluxes into the Arctic Ocean (Woodgate and Peralta-Ferriz, 2021). As a major source of oceanic nutrients (Torres-Valdés et al., 2013), the Bering Strait through flow may also exert a profound effect on ecosystems in the Chukchi Sea, the western Arctic Ocean, and even in outflows in the Canadian Arctic Archipelago (Woodgate, 2018; Woodgate and Peralta-Ferriz, 2021).

The increasing Pacific Inflow Water has changed local hydrographic features in the Arctic Ocean and transported more Pacific-origin species into the Arctic: a process called Pacification (Woodgate, 2018; Polyakov et al., 2020). Studies on the Pacific Arctic Region Pacification have mainly focused on mesozooplankton and phytoplankton communities (Ershova et al., 2015; Wassmann et al., 2015; Hunt et al., 2016; Wang et al., 2018; Xu et al., 2018; Lewis et al., 2020; Wang Y. et al., 2020; Mueter et al., 2021; Zhuang et al., 2021). By analyzing mesozooplankton data from 1946 to 2012, Ershova et al. (2015) found that the distribution of Pacific copepods (*Eucalanus bungii*, *Metridia pacifica*, and *Neocalanus* spp.) in 2012 extend about 5 further north than in 1946. Wang Y. et al. (2020) found that Pacific-origin phytoplankton species can be transported into the Chukchi Sea. These results indicated that the pelagic ecosystem in the Pacific Arctic Region is experiencing rapid Pacification. Despite their important contribution to microzooplankton, there have not been any studies about the pacification of ciliate communities.

Taxonomically, planktonic ciliates belong to phylum Ciliophora, class Spirotrichea, subclass Oligotrichia, and Choreotrichia (Lynn, 2008), and morphologically consist of aloricate ciliate and tintinnid. Planktonic ciliates (belonging to microzooplankton) are primary consumers of pico- (0.2–2 μm) and nano- (2–20 μm) sized plankton and are important food items of metazoans and fish larvae (Stoecker et al., 1987; Dolan et al., 1999; Gómez, 2007). They play an important role in material circulation and energy flow from the microbial food web into the classical food chain (Azam et al., 1983; Pierce and Turner, 1992; Calbet and Saiz, 2005). Furthermore, ciliates have been widely used as a useful bioindicator of different water masses owing to their simple, short life cycle and sensitive response to environmental changes (Kato and Taniguchi, 1993; Kim et al., 2012; Jiang et al., 2013; Wang et al., 2021a, 2022a).

As for planktonic ciliates, Taniguchi (1984) found that aloricate ciliates and tintinnids were dominant taxa in the Bering Sea and Bering Strait, but their abundance showed increasing and decreasing trends from the Bering Sea to Bering Strait, respectively. Subsequent studies found a similar phenomenon and further realized that the Bering Sea, Bering Strait, and Arctic Ocean had their endemic species (Dolan et al., 2014, 2016; Wang et al., 2019). During the summer of 2020, Pacific species (*Salpingella* sp.1) had intruded into the Canada Basin with a higher abundance than Arctic endemic species (Wang et al., 2022a). These previous studies mainly researched ciliate (especially tintinnid) vertical distribution patterns and northward transported trends at a specific time. However, there are still no studies about planktonic ciliates community variations correlated with hydrographic features (temperature, salinity, and Chlorophyll-*a* concentrations) in different years.

As an important trophic link between mesozooplankton and phytoplankton, we hypothesize that the planktonic ciliate community in the Pacific Arctic Ocean is also experiencing rapid Pacification progress under environmental variations induced by global warming. By comparing environmental factors and planktonic ciliate community structure (e.g., abundance proportion of tintinnid to total ciliate, aloricate ciliate size-fraction, tintinnid richness, and latitudinal distribution variations) of 2016 and 2019 in this region, we aim to determine how variations in environmental factors affect ciliate communities. Our results will help monitor changes in the Pacific Arctic Ocean pelagic ecosystem in response to global warming.

## Materials and Methods

### Study Area and Sample Collection

**FIGURE 1:**
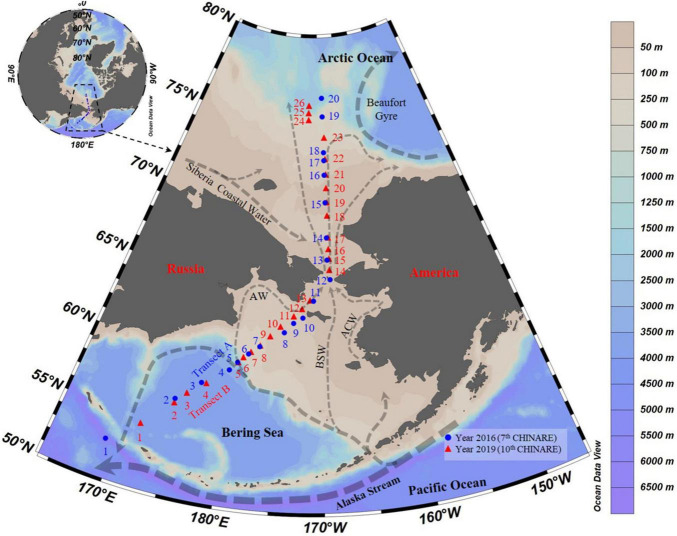
Transects and survey stations from the Bering Sea to the Arctic Ocean in summer of 2016 and 2019. Arrows showed currents following Aksenov et al. (2016), Hunt et al. (2016), Zhong et al. (2019), and Andreev et al. (2020); AW, Anadyr Water; BSW, Bering Shelf Water; ACW, Alaskan Coastal Water.

Sampling was conducted during two cruises performed from July 18 to September 10, 2016 (Transect A), during the 7th Chinese National Arctic Research Expedition aboard *R.V.* “Xuelong,” and from August 24 to September 2, 2019 (Transects B), during the 10th Chinese National Arctic Research Expedition aboard *R.V.* “Xiangyanghong 01” from the Bering Sea to the Arctic Ocean (Figure 1). Water samples were collected at 45 stations (St.) along two transects (Tr.): Tr. A (Sts. 1–20) and B (Sts. 1–26) (Figure 1 and Supplementary Table 1). Stations A1 to A5, A19, A20, B1 to B6, and B24 to B26 were located over depths exceeding 200 m (Supplementary Table 1). We treated A1 to A5, B1 to B6 as the Bering Sea stations, A19, A20, B24 to B26 as the Arctic Ocean stations, and A6 to A18 and B7 to B23 as the Bering Strait stations (depths shallower than 200 m).

Vertical profiles of temperature and salinity were obtained at each station from the surface (3 m) to the bottom (or 200 m, where the bottom is deeper than 200 m) using an SBE911-conductivity-temperature-depth (CTD) unit. Water samples were taken from three to eight depths (from surface to bottom or 200 m depth with stations deeper than 200 m) at each station using 12 L Niskin bottles attached to a rosette wheel of the CTD (sampling points). A total of 251 water samples (1 L) were collected for planktonic ciliate community structure analysis. Samples were fixed with acid Lugol’s (1% final concentration) and stored in darkness at 4°C during the cruise. All the stations were free of sea ice. Chlorophyll *a* (Chl *a*) concentration was determined by filtering 500 mL of seawater through a Whatman GF/F glass fiber filter. Plankton retained on the filter was extracted in 90% (vv^–1^) acetone. Fluorescence was measured according to the Joint Global Ocean Flux Study (JGOFS) protocol (Knap et al., 1996) using a Turner Trilogy fluorometer Model 10.

### Sample Analysis and Species Identification

In the laboratory, water samples were concentrated to ∼200 mL by siphoning off the supernatant after settling the sample for 60 h. This settling and siphoning process was repeated until a final concentrated volume of 50 mL was achieved, which was then settled in two Utermöhl counting chambers (25 mL per chamber) (Utermöhl, 1958) for at least 24 h. Planktonic ciliates were counted using an Olympus IX 73 inverted microscope (100 × or 400 ×) according to the process of Lund et al. (1958) and Utermöhl (1958).

For each species, the size (length, width, and according to shape) of the cell (aloricate ciliate) or lorica (tintinnid, especially length and oral diameter) were measured for at least 20 individuals if possible. Aloricate ciliates were categorized into size-fractions in increments of 10 μm for maximum body length for each individual following Lessard and Murrell (1996), Taylor et al. (2011), and Wang C. F. et al. (2020). The size-fractions were further clustered into small (10–20 μm), medium (20–30 μm), and large (>30 μm) (Sohrin et al., 2010). Tintinnid taxa were identified to the species level according to the size and shape of loricae following Taniguchi (1976), Davis (1977, 1981), Zhang et al. (2012), Dolan et al. (2014, 2017), Li et al. (2016), and Wang et al. (2019, 2022a,b). Because mechanical and chemical disturbance during collection and fixation can detach the tintinnid protoplasm from the loricae (Paranjape and Gold, 1982; Alder, 1999), we included empty tintinnid loricae in cell counts.

### Data Processing

Ciliate volumes were estimated using appropriate geometric shapes (cone, ball, and cylinder). Tintinnid carbon biomass was estimated using the equation:

$$C=V_{i}\times 0.053+444.5\,\left(\text{Verity and Lagdon, 1984}\right).$$

Where *C* (μg C L^–1^) was the carbon and *V*_*i*_ (μm^3^) was the lorica volume. We used a conversion factor of carbon biomass for aloricate ciliates of 0.19 pg/μm^3^ (Putt and Stoecker, 1989). Calculation of ciliate depth-integrated abundance and biomass in water column following Yu et al. (2014). Biogeographically, the classification of tintinnid genera (Neritic, species largely restricted to nearshore waters; Boreal, species restricted to Arctic and Subarctic waters; Cosmopolitan, species distributed widely in the world ocean) was based on Pierce and Turner (1993) and Dolan and Pierce (2013). The threshold for Pacific Inflow Water was 4^°^C as Yamashita et al. (2019) in our results. Data of total surface heat flux (SHF = net solar radiation + net longwave radiation + sensible heat flux + latent heat flux) were obtained from the European Centre for Medium-Range Weather Forecasts (ECMWF).^1^

Horizontal and vertical distribution of environment and ciliate data are presented by ODV (Ocean Data View, Version 5.0, Reiner Schlitzer, Alfred Wegener Institute, Bremerhaven, Germany), Surfer (Version 13.0, Golden Software Inc., Golden, CO., United States), OriginPro 2021 (Version 9.6, OriginLab Corp., United States), and Grapher (Version 12.0, Golden Software Inc., Golden, CO., United States). RELATE analysis was conducted based on Spearman’s correlation between square root-transformed abundance data and log-transformed abiotic parameters (normalized the abiotic parameters, including temperature, salinity, and Chlorophyll-*a*) to explore whether the environment had an effect on organisms, which is a function in PRIMER (Version 6.0, Plymouth Routes in Multivariate Ecological Research). Biota-Environment (BIOENV) analysis was performed based on Spearman’s correlation between log-transformed abiotic parameters and square root-transformed abundance data using PRIMER. The significance for grouping in the environment and ciliate community (aloricate ciliate and tintinnid) was tested by PERMANOVA analysis in PERMANOVA + of PRIMER 6 (Anderson et al., 2008; Jiang et al., 2016). SIMPER (Clarke and Warwick, 1994) analysis was conducted with a criterion of tintinnid dominant species/aloricate ciliate three size-fractions by cutting off for low contributions: 90.00% in 2016 and 2019 using PRIMER.

## Results

### Hydrographic Feature Variations

**FIGURE 2:**
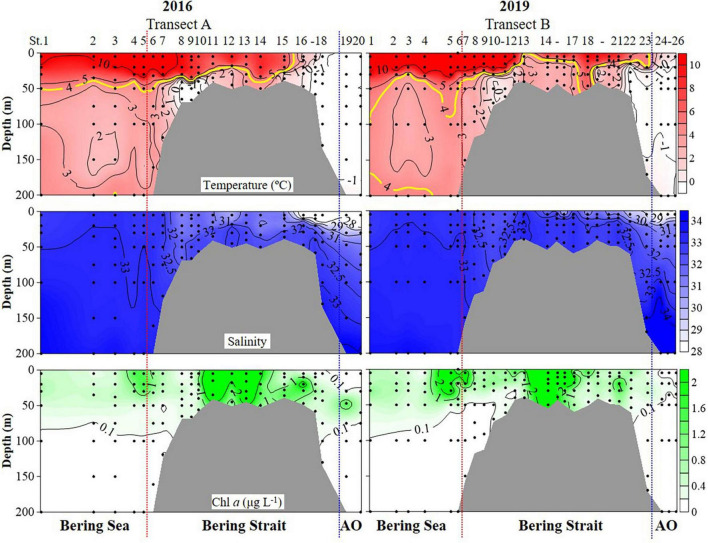
Temperature, salinity, and Chlorophyll *a* (Chl *a*) profiles from surface to bottom (or 200 m). Black dots, sampling points; red dotted line, boundary between the Bering Sea and Bering Strait; blue dotted line, boundary between the Bering Strait and Arctic Ocean (AO).

Hydrographic features (temperature, salinity, and Chlorophyll *a* (Chl *a*) concentrations) showed significant variations during cruises in the summers of 2016 and 2019 (PERMANOVA pseudo-*F* = 2.9832, *P* = 0.043) (Figure 2 and Supplementary Figure 1 and Table 1). Horizontally, temperature continually decreased northward in 2016. While in 2019, the temperature first decreased to St. B13, increased to St. B17, and eventually decreased to the Arctic Ocean (Figure 2). The average temperature in surface layers of 2019 (10.85 ± 0.31°C, 8.22 ± 2.43^°^C, and 0.48 ± 0.43^°^C) were 0.40°, 1.73°, and 1.82^°^C higher than that in 2016 (10.45 ± 0.41°, 6.49 ± 3.67°, and −1.34 ± 0.01^°^C) in the Bering Sea, Bering Strait, and the Arctic Ocean, respectively (Supplementary Figure 1). Vertically, temperature first decreased, then increased to 200 m layers in the Bering Sea in both 2019 and 2016, while the average temperature from 50 to 200 m layers in 2019 was higher than that in 2016. In the Bering Strait, the temperature decreased from surface to bottom in both 2019 and 2016. In the Arctic Ocean, temperature showed almost no change from surface to 200 m depth in 2016. However, temperature first decreased from surface to 50 m layers, then increased to 200 m layers in 2019 (Figure 2 and Supplementary Figure 1).

**TABLE 1:** Results of PERMANOVA based on Euclidean distance matrices derived from log-transformed environmental data between 2016 and 2019.

PERMANOVA table of results				

	df	MS	Pseudo-*F*	*P*
Groups	1	8.8788	2.9832	0.043
Residual	248	2.9763		
Total	249			

Horizontally, salinity continually decreased northward in both 2016 and 2019, while the average salinity in surface layers of 2019 (32.88 ± 0.11, 31.54 ± 1.24, and 28.25 ± 0.28) were 0.06, 1.08, 0.76 higher than that in 2016 (32.82 ± 0.13, 30.46 ± 2.03, and 27.49 ± 0.70) in the Bering Sea, Bering Strait, and the Arctic Ocean, respectively (Figure 2 and Supplementary Figure 1). Vertically, salinity increased from the surface to 200 m layers or bottom in the Bering Sea, Bering Strait, and Arctic Ocean (Figure 2). Except for 200, 100, and 50 m layers, the average salinity value in other layers in 2019 was higher than that in 2016 (Supplementary Figure 1).

Chl *a* showed similar distribution characteristics in both 2016 and 2019, but there were still several differences (Figure 2 and Supplementary Figure 1). Horizontally, Chl *a* increased from the Bering Sea to Bering Strait, then decreased northward in both 2016 and 2019, while the average Chl *a* in surface layers of 2019 (0.96 ± 1.49 μg L^–1^, 2.43 ± 4.36 μg L^–1^, and 0.40 ± 0.01 μg L^–1^) were higher than that in 2016 (0.78 ± 0.60 μg L^–1^, 1.28 ± 1.51 μg L^–1^, and 0.04 ± 0.01 μg L^–1^) in the Bering Sea, Bering Strait, and the Arctic Ocean, respectively (Figure 2 and Supplementary Figure 1). For vertical distribution, Chl *a* decreased from surface to 200 m or bottom generally in the Bering Sea and Bering Strait in both 2016 and 2019. While in the Arctic Ocean, deep Chl *a* maximum (DCM) layers in 2019 (40 m) were shallower than that in 2016 (50 m) and the average Chl *a* in DCM of 2019 (0.14 ± 0.12 μg L^–1^) was lower than that in 2016 (0.93 ± 0.74 μg L^–1^) (Figure 2 and Supplementary Figure 1).

The total surface heat flux in the Pacific Arctic Region showed that the ocean gained heat from air both in August and September, but the heat from the atmosphere to the ocean in 2019 was lower than that in 2016 in most stations of our study area (Supplementary Figure 2).

### Variations in Planktonic Ciliate Abundance and Biomass in 2016 and 2019

**FIGURE 3:**
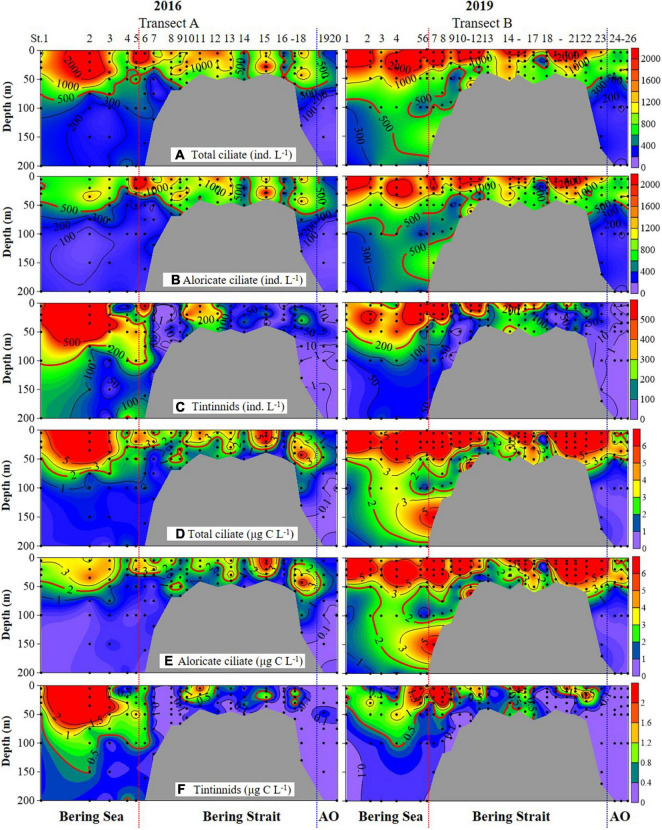
Vertical distribution of total ciliate, aloricate ciliate, and tintinnid abundance **(A–C)** and biomass **(D–F)** from surface to bottom (or 200 m). Black dots, sampling depths; red dotted line, boundary between the Bering Sea and Bering Strait; blue dotted line, boundary between the Bering Strait and Arctic Ocean (AO).

Ciliate abundance and biomass generally decreased northward (from the Bering Sea to the Arctic Ocean) in both 2016 and 2019, with some significant differences (Figure 3 and Supplementary Figure 3). PERMANOVA tests indicated significant differences between 2 years of aloricate ciliate (pseudo-*F* = 17.272, *P* = 0.001) and tintinnid (pseudo-*F* = 9.2666, *P* = 0.001) abundance data (Table 2). In the Bering Sea, ciliate high abundance (total ciliate and aloricate ciliate: ≥ 500 ind. L^–1^, tintinnids: 200 ind. L^–1^) and biomass (total ciliate and aloricate ciliate: ≥ 2 μg C L^–1^, tintinnids: 0.5 μg C L^–1^) mainly occurred in upper 100 m in 2019, while these values mainly appeared in upper 50 m in 2016 (Figure 3). Although vertical distribution patterns of ciliate abundance and biomass were the same in both 2016 and 2019, the highest average total abundance (2025.67 ± 1628.80 ind. L^–1^) and biomass (9.66 ± 4.80 μg C L^–1^) at 20 m in 2019 were 1.21 and 1.79 folds higher than that in 2016 (1679.20 ± 1034.85 ind. L^–1^; 5.39 ± 3.87 μg C L^–1^) (Supplementary Figure 3). The proportion of tintinnid abundance and biomass to total ciliate in 2019 (18.68 ± 3.29%, 12.75 ± 2.46%) was much lower than that in 2016 (41.79 ± 8.96%, 46.15 ± 10.33%) (Supplementary Figure 3).

**TABLE 2:** Results of PERMANOVA based on Bray Curtis similarity matrices derived from Square root-transformed abundance data of aloricate ciliates and tintinnids between 2016 and 2019.

	df	MS	Pseudo-*F*	*P*
**PERMANOVA between aloricate ciliates**				
Groups	1	11,004	17.272	0.001
Residual	248	637.13		
Total	249			
**PERMANOVA between tintinnids**				
Groups	1	28,998	9.2666	0.001
Residual	215	3129.3		
Total	216			

In the Bering Strait, ciliate abundance and biomass decreased from surface to bottom in both 2016 and 2019, while the highest average total abundance (2224.92 ± 1131.25 ind. L^–1^) and biomass (11.02 ± 8.56 μg C L^–1^) at 5 m in 2019 were 2.11 and 4.11 folds higher than that in 2016 (1053.29 ± 692.03 ind. L^–1^; 2.68 ± 2.08 μg C L^–1^) (Figure 3 and Supplementary Figure 3). The proportion of tintinnid abundance and biomass to total ciliate in 2019 (10.53 ± 4.40%, 9.03 ± 4.13%) was lower than that in 2016 (14.88 ± 5.42%, 17.69 ± 4.88%) (Supplementary Figure 3).

In the Arctic Ocean, ciliate abundance and biomass increased from surface to DCM layers, then decreased to 200 m, but the highest average total abundance (876.67 ± 277.29 ind. L^–1^) and biomass (3.71 ± 1.15 μg C L^–1^) in DCM layers of 2019 were 1.73 and 2.81 folds higher than in 2016 (507.50 ± 177.48 ind. L^–1^; 1.32 ± 0.63 μg C L^–1^) (Figure 3 and Supplementary Figure 3). Tintinnid had low abundance and biomass in both 2019 and 2016, while the proportion of tintinnid abundance and biomass to total ciliate in 2019 (0.38 ± 0.25%, 0.31 ± 0.30%) were lower than that in 2016 (3.18 ± 3.02%, 6.93 ± 6.20%) (Supplementary Figure 3).

Latitudinally, ciliate integrated abundance and biomass increased from the Bering Sea to Bering Strait, then decreased to the Arctic Ocean in both 2016 and 2019. However, those values in the Bering Sea (0.86 ± 0.25 × 10^6^ ind. m^–2^, 3.09 ± 0.93 mg C m^–2^), Bering Strait (1.24 ± 0.54 × 10^6^ ind. m^–2^, 6.20 ± 3.81 mg C m^–2^), and Arctic Ocean (0.29 ± 0.06 × 10^6^ ind. m^–2^, 0.99 ± 0.15 mg C m^–2^) in 2019 were higher than that in 2016 (Bering Sea 0.59 ± 0.16 × 10^6^ ind. m^–2^, 1.86 ± 0.87 mg C m^–2^; Bering Strait 0.82 ± 0.34 × 10^6^ ind. m^–2^, 2.14 ± 1.39 mg C m^–2^; Arctic Ocean 0.20 ± 0.06 × 10^6^ ind. m^–2^, 0.46 ± 0.13 mg C m^–2^), respectively (Supplementary Figure 4 and Supplementary Table 1).

### Aloricate Ciliate Size-Fraction Abundance and Abundance Proportion Variations

**FIGURE 4:**
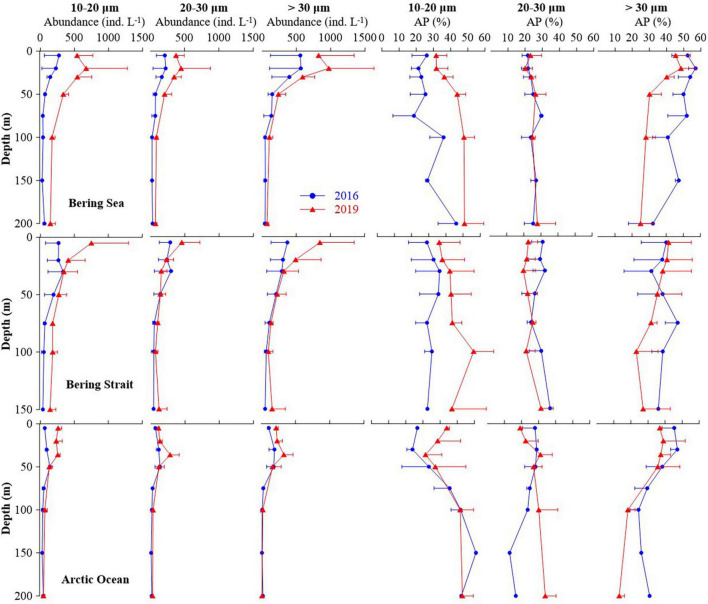
Average abundance and abundance proportion of each aloricate ciliate size-fraction at each layers in the Bering Sea, Bering Strait, and Arctic Ocean.

Aloricate ciliates were the main contributors to the observed increase in ciliate abundance in the summer of 2019, compared with 2016 (Figure 4 and Supplementary Figure 3). The average abundance and abundance proportion of aloricate ciliate small (10–20 μm) size-fraction in the upper 50 m layers of the Bering Sea, Bering Strait, and the Arctic Ocean in 2019 were higher than that in 2016. The average abundance of large (> 30 μm) size-fraction in the upper 50 m layers of three seas in 2019 was higher than that in 2016, but the average abundance proportion was lower in the Bering Sea and Arctic Ocean (Figure 4). For integrated abundance of small, medium (20–30 μm), and large size-fraction groups, an increase occurred in the Bering Sea, Bering Strait, and the Arctic Ocean in 2019 compared with 2016, respectively. In 2016, the most abundant group was the large size-fraction in the Bering Sea (50.77 ± 3.31%), Bering Strait (36.88 ± 10.80%), and Arctic Ocean (39.19 ± 5.05%). While in 2019, the small size-fraction was the most abundant (Bering Sea 39.94 ± 3.71%, Bering Strait 38.87 ± 8.82%, and Arctic Ocean 39.70 ± 11.12%) (Supplementary Figure 5).

### Tintinnid Composition and Latitudinal Distribution Variations in 2016 and 2019

A total of 49 tintinnid species belonging to 15 genera were identified (Supplementary Figure 6 and Supplementary Table 2). Tintinnid species richness in 2019 (45 species) was higher than that in 2016 (35 species). All tintinnid species were classified into abundant and rare species according to their maximum abundance (A_max_) and occurrence frequency (OF). We defined abundant species as those with A_max_ ≥ 100 ind. L^–1^ and OF ≥ 40% (Table 1). Other species were defined as rare species (Supplementary Table 2).

**FIGURE 5:**
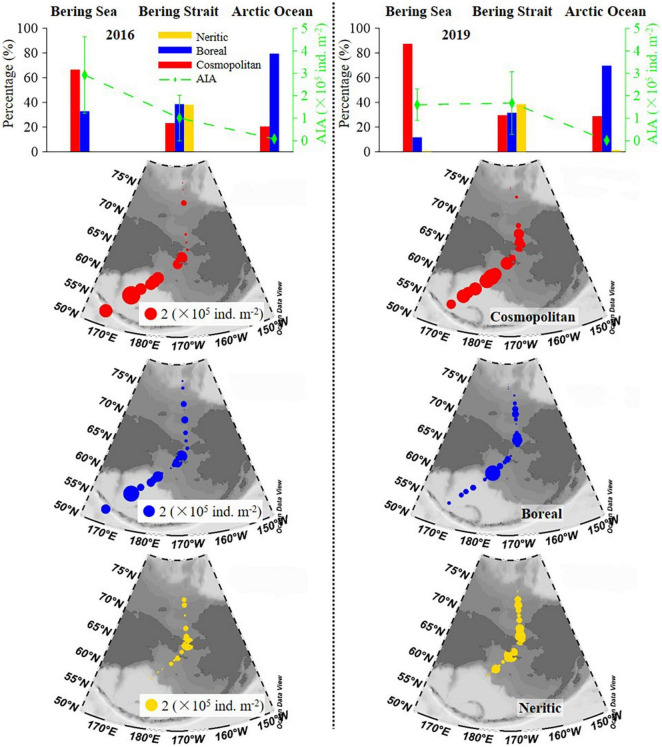
Latitudinal variation of cosmopolitan, neritic, and boreal tintinnid (average) integrated abundance and its percentage. AIA, average integrated abundance.

Geographical distribution trends of the average integrated abundance of tintinnid were different in 2016 and 2019. In 2016, this value gradually decreased from the Bering Sea to the Arctic Ocean, while in 2019, the average integrated abundance in the Bering Sea was similar to that in the Bering Strait, then decreased sharply to the Arctic Ocean (Figure 5 and Supplementary Table 3). Oceanic genera (cosmopolitan and boreal) were distributed in all three seas. Neritic genera (neritic) mainly occurred in the Bering Strait (Figure 5).

The distribution trend of average integrated abundance and the relative proportion of each biogeographical category were similar in both 2016 and 2019, while there were still some differences (Figure 5 and Supplementary Table 3). In the Bering Sea and the Arctic Ocean, an average integrated abundance of cosmopolitan and boreal genera in 2019 was lower than in 2016, respectively. But in the Bering Strait, cosmopolitan and boreal genera were 2.03 and 1.15 folds higher than in 2016, respectively. The average integrated abundance of neritic genera in 2019 was also higher than in 2016 (Supplementary Table 3). As for average integrated abundance proportions in the Bering Sea, Bering Strait, and the Arctic Ocean, cosmopolitan genera in 2019 were 20.72, 6.45, and 8.30% higher than in 2016, respectively. However, boreal genera in 2019 were lower than in 2016, respectively (Supplementary Table 3).

**FIGURE 6:**
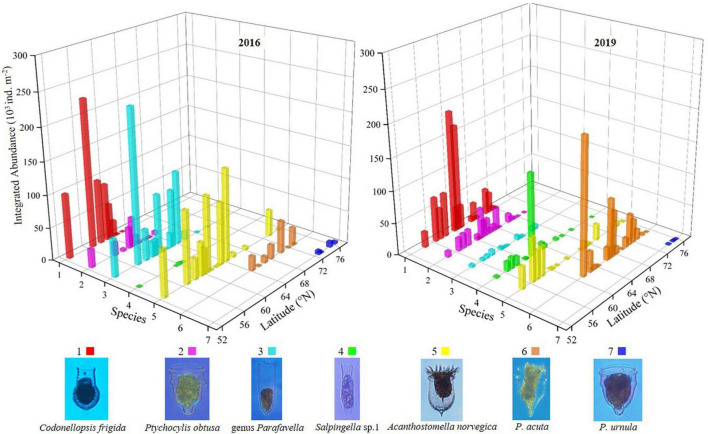
Latitudinal distribution variation of dominant oceanic tintinnid integrated abundance.

The latitudinal distribution of abundant oceanic tintinnids was different from the Bering Sea to the Arctic Ocean between 2 years (Figure 6 and Supplementary Figure 7). *Codonellopsis frigida*, *Ptychocylis obtusa*, genus *Parafavella*, *Salpingella* sp.1, and *Acanthostomella norvegica* were abundant in the Bering Sea in 2016 or 2019. Among them, the distribution of *C. frigida*, *P. obtusa*, and *Salpingella* sp.1 in 2019 have expanded north to 68.2°N, 69.5°N, and 69.5°N, respectively, which were 5.9, 5.2, and 8.8 degrees further north of where they occurred in 2016. While genus *Parafavella* distributed southward in 2019 (66.9°N) compared to 2016 (70.3°N). *P. acuta* was an abundant oceanic tintinnid in the Bering Strait, and its distribution in 2019 was wider than in 2016. In the Arctic Ocean, the distribution range of oceanic tintinnid *P. urnula* was narrower in 2019 than in 2016 (Figure 6 and Supplementary Figure 7).

### Relationship Between Ciliates and Environmental Factors

Correlations between ciliates (aloricate ciliates and tintinnids) and environmental variables (temperature, salinity, and Chl *a*) were different. The routine RELATE test showed that there were significant correlations between changes in environmental variables and tintinnids (*Rho* = 0.318, *P* = 0.001), while the impact of the environment on aloricate ciliates (*Rho* = 0.126, *P* = 0.001) was smaller. In addition, the multivariate biota-environment (BIOENV) analysis was conducted to select the combination of environmental factors (temperature, salinity, and Chl *a*) that have the greatest impact on the ciliate community structure. The analysis showed that the best match with tintinnids was a combination of temperature and salinity (*Rho* = 0.430, *P* = 0.01), while aloricate ciliates were most impacted by temperature alone (*Rho* = 0.163, *P* = 0.01) (Table 3).

**TABLE 3:** Summary of results from BIOENV (biota-environment) analysis showing the best matches of combinations of environmental variables with variations in aloricate ciliate and tintinnid abundance.

Rank	Best combination of variables	Correlation coefficient
**Aloricate ciliate**		
1	T	0.163
2	T, Chl *a*	0.152
3	T, SAL, Chl *a*	0.126
4	T, SAL	0.125
5	Chl *a*	0.121
6	SAL, Chl *a*	0.074
7	SAL	0.019
**Tintinnid**		
1	T, SAL	0.430
2	T	0.369
3	T, SAL, Chl *a*	0.318
4	SAL	0.276
5	SAL, Chl *a*	0.241
6	SAL, Chl *a*	0.148
7	Chl *a*	−0.021

*T, Temperature; SAL, Salinity; Chl a, Chlorophyll a.*

For the three aloricate ciliate size-fractions (10–20 μm, 20–30 μm, and > 30 μm) in the Bering Sea, Bering Strait, and the Arctic Ocean, SIMPER analysis revealed that the small size-fraction was more dominant in 2019 (contrib% = 38.37) than that in 2016 (contrib% = 33.52) (Table 4). As for oceanic abundant tintinnid in the Bering Sea (genus *Parafavella*, *C. frigida*, *P. obtusa*, and *A. norvegica*) and Bering Strait (*P. acuta*), SIMPER analysis indicated that the composition of Bering Sea dominant tintinnid species changed significantly between 2016 and 2019. In 2016, genus *Parafavella* (Contrib% = 32.71) dominated, then followed by *A. norvegica* (Contrib% = 27.72), *C. frigida* (Contrib% = 15.54), and *P. obtusa* (Contrib% = 10.05). While in 2019, *C. frigida* (Contrib% = 34.63) became the most dominant species and the contribution rate of the genus *Parafavella* was the lowest (<9.68%) (Table 4). No abundant oceanic species were detected in the Arctic Ocean in either 2016 or 2019.

**TABLE 4:** Results from SIMPER analysis based on Bray Curtis similarity showing community composition of aloricate ciliate and tintinnid whose cumulative contribution rate was higher than 90% in 2016 and 2019, respectively.

Aloricate ciliate				Tintinnids			
Size-fraction (μm)	Av.Abund	Contrib%	Cum.%	Species	Av.Abund	Contrib%	Cum.%

**2016**				**2016**			
>30	14.17	35.88	35.88	Genus *Parafavella*	3.87	32.71	32.71
10–20	12.44	33.52	69.39	*Acanthostomella norvegica*	3.56	27.72	60.43
20–30	11.84	30.61	100.00	*Codonellopsis frigida*	3.33	15.54	75.97
				*Ptychocylis obtusa*	1.72	10.05	86.02
				*P. acuta*	0.86	8.02	94.04

**2019**				**2019**			

10–20	18.29	38.37	38.37	*C. frigida*	3.85	34.63	34.63
>30	18.20	33.09	71.46	*P. acuta*	2.26	25.69	60.32
20–30	14.02	28.54	100.00	*P. obtusa*	2.09	17.41	77.73
				*A. norvegica*	1.93	12.58	90.32

### Temperature-Salinity-Plankton Diagrams for Abundant Tintinnid Species

**FIGURE 7:**
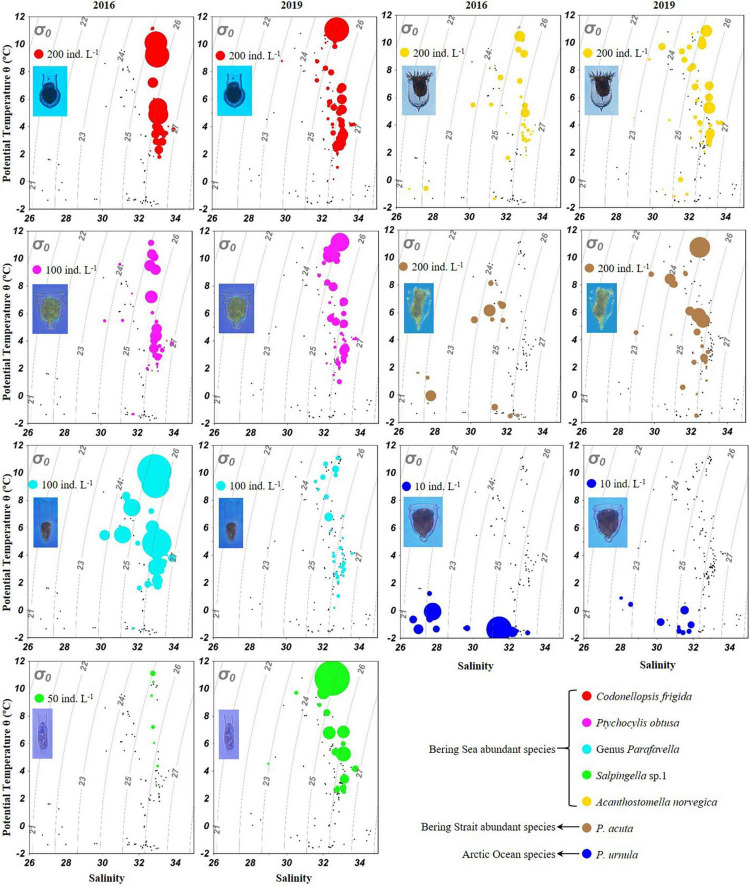
Temperature-salinity-plankton diagrams for abundant oceanic tintinnids in 2016 and 2019.

Temperature-salinity-plankton diagrams showed that seven abundant tintinnid species had different temperature and salinity ranges (Figure 7). In the Bering Sea, high abundance (≥ 100 ind. L^–1^) of *C. frigida*, *P. obtusa*, and *A. norvegica* mainly occurred in relatively higher temperatures (2.0–11.8^°^C) but narrower salinity range (32.7–33.3) in both 2016 and 2019. In contrast, the genus *Parafavella* had a narrower salinity range and *Salpingella* sp.1 had a wider temperature range in 2019 than in 2016, respectively. In the Bering Strait and the Arctic Ocean, *P. acuta* had a wider temperature range and *P. urnula* had a narrower salinity range in 2019 than in 2016, respectively (Figure 7).

## Discussion

The Pacific Inflow Water (PIW) brings heat and fresh water to the Arctic Ocean in summer, resulting in sea ice melt and renewal of the nutrients, and supporting Arctic ecosystems (Grebmeier et al., 2006; Woodgate and Peralta-Ferriz, 2021). In recent years, significant warming in the annual mean temperatures of the PIW has been observed (Woodgate and Peralta-Ferriz, 2021). The atmosphere has significant effects on the heat budget of upper waters in the Pacific Arctic Region. After calculating the total surface heat flux in the Pacific Arctic Region, we found that the heat from the atmosphere to the ocean in 2019 was lower than that in 2016 (Supplementary Figure 2). Therefore, we conclude that the higher sea temperature in 2019 was due to warm advection from PIW, bringing heat to the Arctic Ocean.

Planktonic ciliates, as an important component of microzooplankton, have been extensively investigated for their important ecological roles in the Pacific Arctic Region (e.g., Jiang et al., 2015; Dolan et al., 2016, 2021; Li et al., 2016; Xu et al., 2018a,b; Wang et al., 2019, 2022b; Wang C. F. et al., 2020). However, studies related to ciliate Pacification are still scant. After comparing hydrographic features at similar locations and sampling times in 2016 and 2019, we found warmer Pacific Inflow Water in 2019 (Supplementary Figure 1), and propose potential identification of ciliate Pacification characteristics.

### Rapid Pacification Progress of Ciliates in the Pacific Arctic Region

The abundance proportion of tintinnids to total ciliates has been described in tropical, subtropical, and polar seas (Sherr et al., 1997; Yang et al., 2004; Gómez, 2007; Sohrin et al., 2010; Wang et al., 2019, 2021b; Wang C. F. et al., 2020). In the tropical West Pacific, North Pacific, and the Arctic Ocean, average abundance proportions of tintinnids to total ciliates tend to be about 0–10% (Sohrin et al., 2010; Wang et al., 2019, 2021b; Wang C. F. et al., 2020), 10–20% (Gómez, 2007; Sohrin et al., 2010; Wang et al., 2021a), and <2% (Sherr et al., 1997; Wang C. F. et al., 2020), respectively. The highest value was shown to occur in the Bering Sea, where it reaches ∼50% (Taniguchi, 1984). Our results showed that this value in 2019 (18.68 ± 3.29%) was much lower than in 2016 (41.79 ± 8.96%), indicating that warmer and more saline Bering Sea waters result in a similar abundance proportion of tintinnids to total ciliates to that found in the North Pacific (Sohrin et al., 2010; Wang et al., 2021a). The warm Alaska Stream was the main current and transported plankton from the North Pacific to the Bering Sea (Andreev et al., 2020). Therefore, we speculated that the abundance proportion of tintinnids to total ciliates in the Bering Sea will be closer to that in the North Pacific (beginning of the Pacification).

Abundance proportions of different aloricate ciliate size-fractions have rarely been reported in the Pacific Arctic Region. In the tropical West Pacific and North Pacific (Yang et al., 2004; Wang et al., 2021a,b), average abundance proportions of small aloricate ciliate (10–20 μm) to total ciliates ranged from 38 to 50% and this size-fraction was the dominant group at each depth in most stations. While in the Bering Sea, dominance shifted to the large (>30 μm) size-fraction group (Wang C. F. et al., 2020). Although our results showed that the average abundance of the large size-fraction group increased in 2019 compared to 2016 (Figure 4), the average integrated abundance and the proportion of dominant groups changed to small size-fraction (Supplementary Figure 4). A similar phenomenon was previously observed in the North Pacific (Yang et al., 2004; Wang et al., 2021a). With rapid Pacification progress, aloricate ciliate small size-fraction might be more abundant in the Pacific Arctic Region in the future.

The latitudinal diversity gradient in tintinnids appears to be closely related to temperature over a wide variety of time scales (Yasuhara et al., 2012; Dolan et al., 2016), suggesting that temperature has a preponderant role in determining species richness. These studies also showed a decrease in tintinnid richness from the tropical to polar seas (Dolan et al., 2016; Wang C. F. et al., 2020). In our study, tintinnid richness in warmer waters was higher in 2019 than in 2016. This phenomenon was consistent with Yasuhara et al. (2012), Dolan et al. (2016), and Wang C. F. et al. (2020). In addition, new tintinnid species belonging to the cosmopolitan genera which we detected in the Bering Sea in 2019 also appeared in the North Pacific (Li et al., 2021; Wang et al., 2021a). Therefore, we speculate that those new species originating from the North Pacific might be transported by the Alaska Stream (Springer et al., 1996; Andreev et al., 2020).

There have been numerous studies on tintinnid horizontal and vertical distribution (Taniguchi, 1984; Dolan et al., 2016; Li et al., 2016; Xu et al., 2018a,b; Wang et al., 2019), but there is limited information on tintinnid northward transportation. As for the Bering Sea species, *Salpingella* sp.1, *C. frigida*, and *P. obtusa* were reported to successively disappear with a northward progression and did not pass the Bering Strait (Taniguchi, 1984; Li et al., 2016; Wang et al., 2019). However, in our results, those species extended further north to south side of the Chukchi Sea in 2019. The Bering Strait species (*P. acuta*) also extended further north, and the Arctic species (*P. urnula*) distribution range was narrower in 2019 than in 2016. We conclude that stronger Pacific Inflows in 2019 further alter the hydrographic feature of the Chukchi Sea and Arctic Ocean (Woodgate and Peralta-Ferriz, 2021), which will eventually become suitable for the Bering Sea and Bering Strait species to live in, simultaneously reducing the living space of Arctic native species.

### Possible Response of Ciliate to Ongoing Global Warming

In the Pacific Arctic Region, the PIW carries more warm water into the Arctic Ocean in recent years. From 1990 to 2019, Woodgate and Peralta-Ferriz (2021) reported increasing northward flow (0.010 ± 0.006 Sv/yr) and annual mean temperatures (0.05 ± 0.02^°^C/yr), with faster change (∼0.1^°^C/yr) in warming (June/July) and cooling (October/November) months, which were 2°–4^°^C above climatology. The maximum temperature of the Pacific Water Layer increased by ∼0.5^°^C between 2009 and 2013 in the Canada Basin (Timmermans et al., 2014), with a doubling in integrated heat content from 1987 to 2017 (Timmermans et al., 2018). From 2001 to 2014, heat transport associated with Bering Strait inflow increased by 60%, from around 10 TW in 2001 to 16 TW in 2014 (due to increase in both volume flux and temperature) (Woodgate, 2018), which further alter the hydrographical environment of the Arctic Ocean.

Previous studies have shown that macrozooplankton abundance and biomass increase significantly in warmer waters in recent years compared to historical studies (Ershova et al., 2015; Xu et al., 2018; Kim et al., 2022). During each August from 2016 to 2020, macrozooplankton abundance was highest in the Bering Strait with higher water temperature (Kim et al., 2022). As important food items of macrozooplankton, we speculate that higher ciliate abundance and biomass in warmer waters of 2019 might be the main reason for higher macrozooplankton abundance.

In the Pacific Arctic Region, Pacific-origin tintinnids were transported from the Bering Sea to the Arctic Ocean mainly in waters > 4^°^C (Li et al., 2016; Wang et al., 2019, 2022b). For this reason, they probably could not survive in cold Arctic waters (<0^°^C). This phenomenon does not apply completely to all tintinnids. For example, *Salpingella* sp.1 was first recorded in the northwest Pacific and mainly lived in water temperature > 2^°^C in 2014 (Li et al., 2016) and our results. In 2020, this species was transported into the warmer Pacific Summer Water (compared to 2016, Wang et al., 2019) with a temperature range from −0.3° to 0.9^°^C in the Canada Basin of the Arctic Ocean (Wang et al., 2022a). Although our present study did not find PIW sink into the subsurface layers of the Canada Basin, we speculate that, given sustained intrusion trends of warmer waters, more Pacific-origin tintinnids will be found in the future Arctic Ocean. Comparable studies in the Atlantic Gateway are needed to discover whether Atlantic-origin species are being similarly transported into the High Arctic.

The size-fraction for aloricate ciliate or lorica oral diameter (LOD) for tintinnid is related to its preferred food item size, for example, the preferred food item for a tintinnid is about 25% of the LOD (Dolan, 2010). Our results showed a clear increase in abundance and biomass of aloricate ciliate of small size-fraction in warmer Pacific Inflows. This phenomenon revealed that the preferred food item size for aloricate ciliate is getting smaller, and this was consistent with the decreasing trend of phytoplankton size classes (Li et al., 2009; Zhuang et al., 2021). We hypothesize that, as rapid Arctic Pacification progresses, more aloricate ciliate small size-fraction and Pacific-origin tintinnids (belonging to cosmopolitan genera) may be transported into the Arctic Ocean by increasing warm Pacific Inflow Water in the future. Our results only present a “snapshot” phenomenon about ciliate Pacification in 2016 and 2019. Further investigations in the Arctic Ocean are needed to test our hypothesis.

## Conclusion

The present study reported planktonic ciliate community structure variations, relationship with environmental factors in similar locations, and sampling time in the Pacific Arctic Region in 2016 and 2019. In 2019, both temperature and salinity were higher than in 2016, which increased both total ciliate and aloricate ciliate abundance and biomass and a decrease for tintinnids. More aloricate ciliate small size-fraction and Pacific-origin tintinnids (belonging to cosmopolitan genera) occurred in warmer and more saline waters of the Pacific Arctic Region in 2019, and community structure characteristics were more similar to the North Pacific, which suggested the rapid Pacification of Arctic microzooplankton. Multivariate correlation analysis between ciliate communities and environmental variables revealed that temperature has a preponderant role in determining both aloricate ciliate and tintinnid composition.

## Data Availability Statement

The original contributions presented in this study are included in the article/Supplementary Material, further inquiries can be directed to the corresponding author.

## Author Contributions

CW: tintinnid taxonomy and counting, data analysis, and writing—original draft. MY: data analysis. YH and ZX: field sampling and writing—original draft. YZ and TX: conceptualization. WZ: field sampling, conceptualization, and writing—original draft. All authors contributed to the article and approved the submitted version.

## Conflict of Interest

The authors declare that the research was conducted in the absence of any commercial or financial relationships that could be construed as a potential conflict of interest.

## Publisher’s Note

All claims expressed in this article are solely those of the authors and do not necessarily represent those of their affiliated organizations, or those of the publisher, the editors and the reviewers. Any product that may be evaluated in this article, or claim that may be made by its manufacturer, is not guaranteed or endorsed by the publisher.

## Footnotes

1
https://www.ecmwf.int/en/forecasts/datasets/reanalysis-datasets/era5
