# Supplementary material for: Hydrographic Feature Variation Caused Pronounced Differences in Planktonic Ciliate Community in the Pacific Arctic Region in the Summer of 2016 and 2019
Source: Front Microbiol. 2022 Jun 9;13:881048. doi: 10.3389/fmicb.2022.881048 (PMC9221986; doi:10.3389/fmicb.2022.881048)
Supplement: Supplementary file 1 [file Presentation_1.pdf]

Supplemental material figures

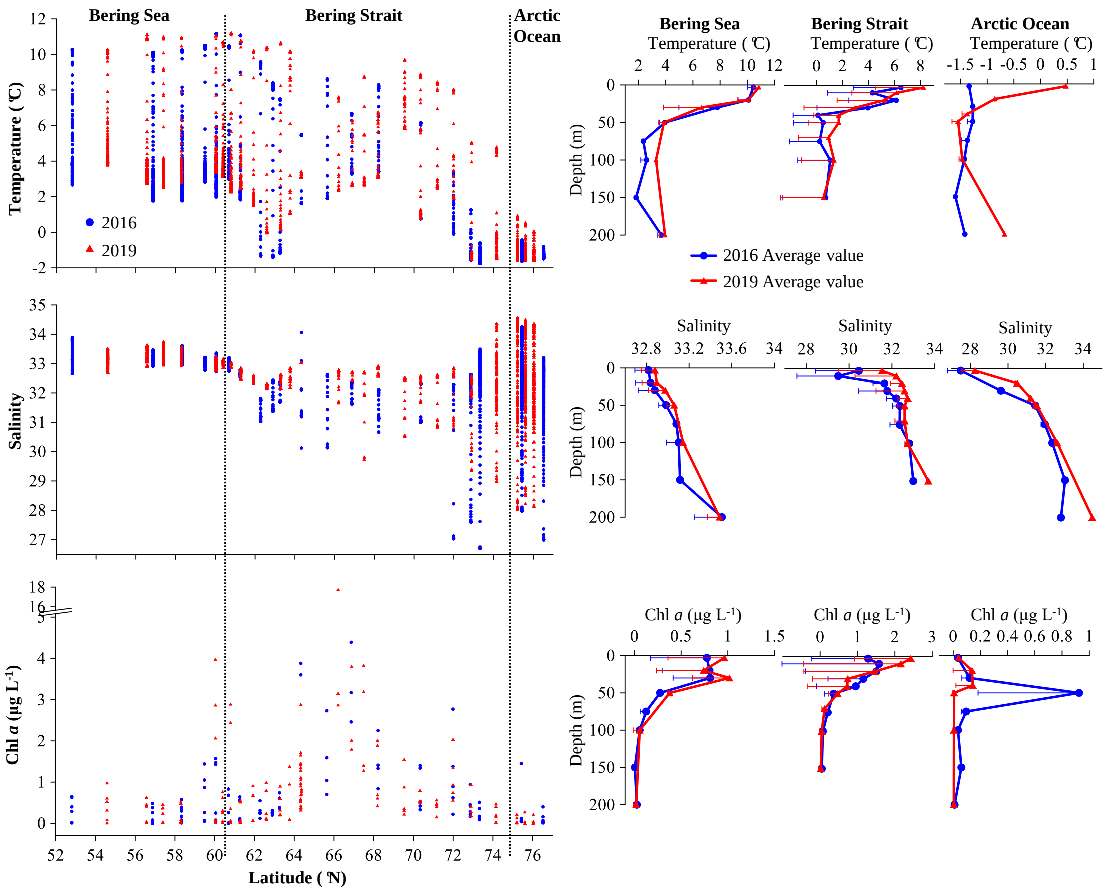

Supplementary Figure 1. Latitudinal and vertical distribution of temperature, salinity and Chl *a* concentrations (Chl *a*) from surface to 200 m from the Bering Sea to the Arctic Ocean.

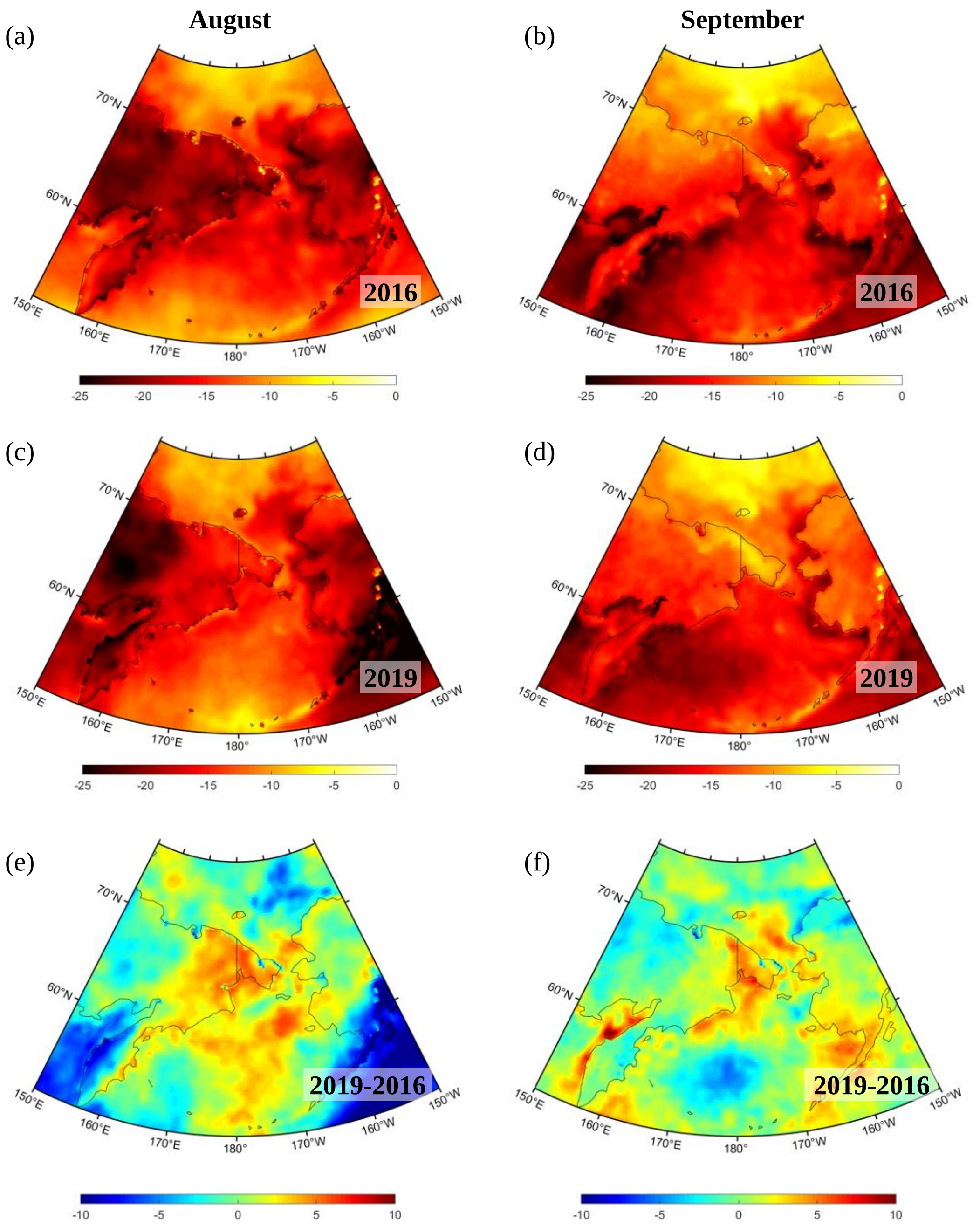

Supplementary Figure 2. The lateral distributions of total surface heat flux (SHF) in the Bering Sea, Bering Strait and Chukchi Sea. SHF in August 2016 (a), September 2016 (b), August 2019 (c) and (d) September 2019 (Unit: 10^6 J•m^{-2}). (e), the difference between (c) and (a), which equals SHF in August 2019 minus SHF in August 2016. (f), the difference between (d) and (b). Data resource: https://www.ecmwf.int/en/forecasts/datasets/reanalysis-datasets/era5.

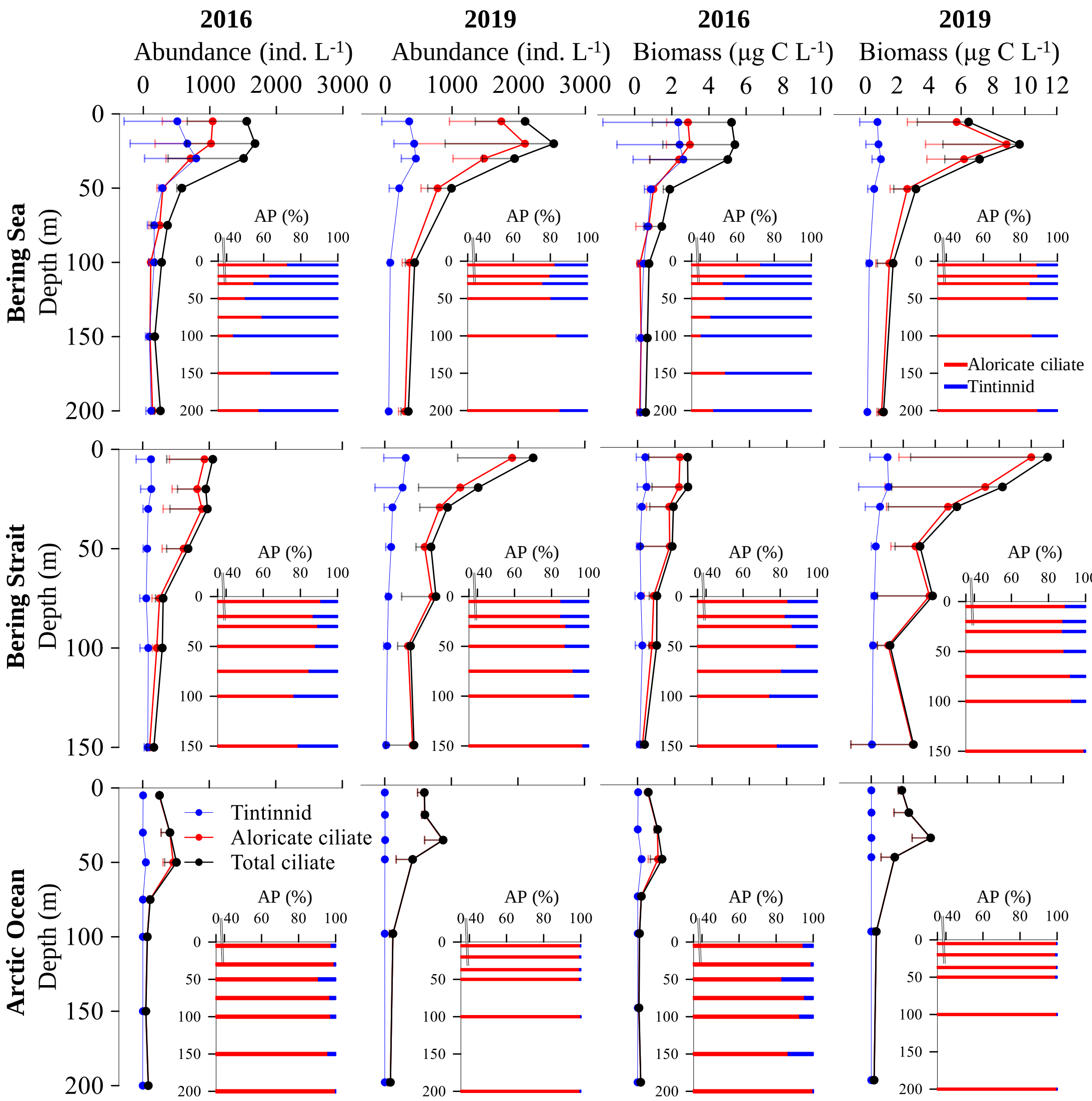

Supplementary Figure 3. Vertical distribution of average planktonic ciliate (aloricate ciliate and tintinnid) abundance and biomass, and abundance proportion (AP) of two groups to total ciliate in every sampling layers.

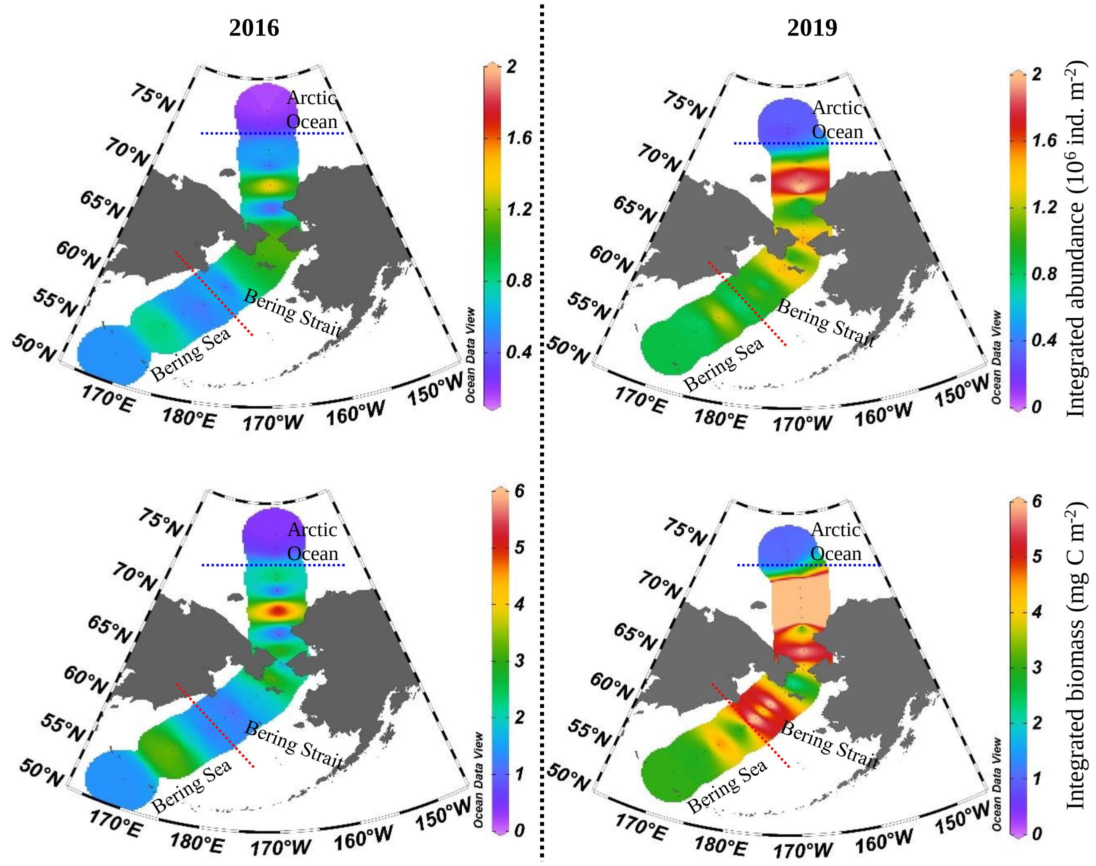

Supplementary Figure 4. Latitudinal variation of ciliate integrated abundance and biomass from the Bering Sea to the Arctic Ocean.

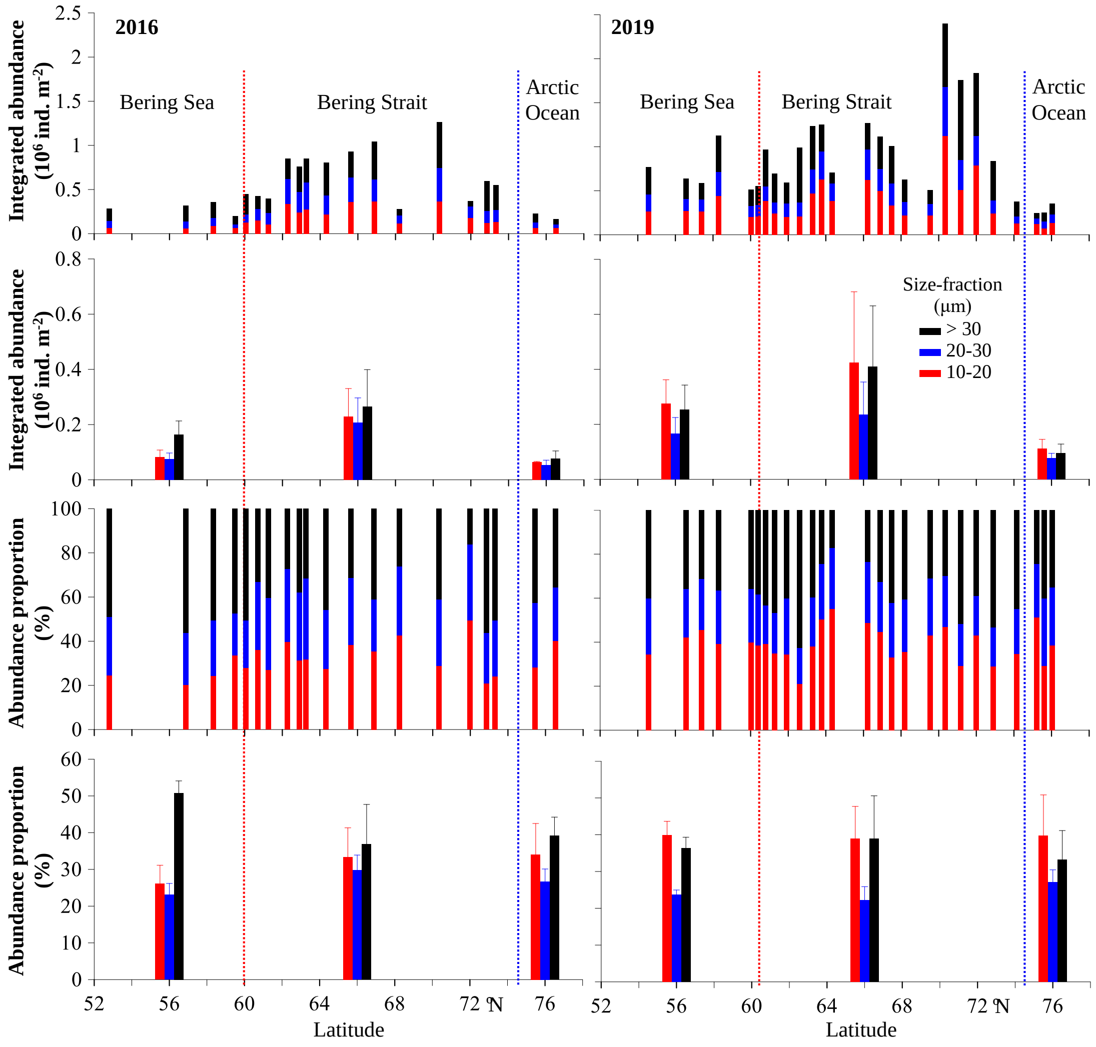

Supplementary Figure 5. Latitudinal variation of three aloricate ciliate size-fraction integrated abundance and its abundance proportion.

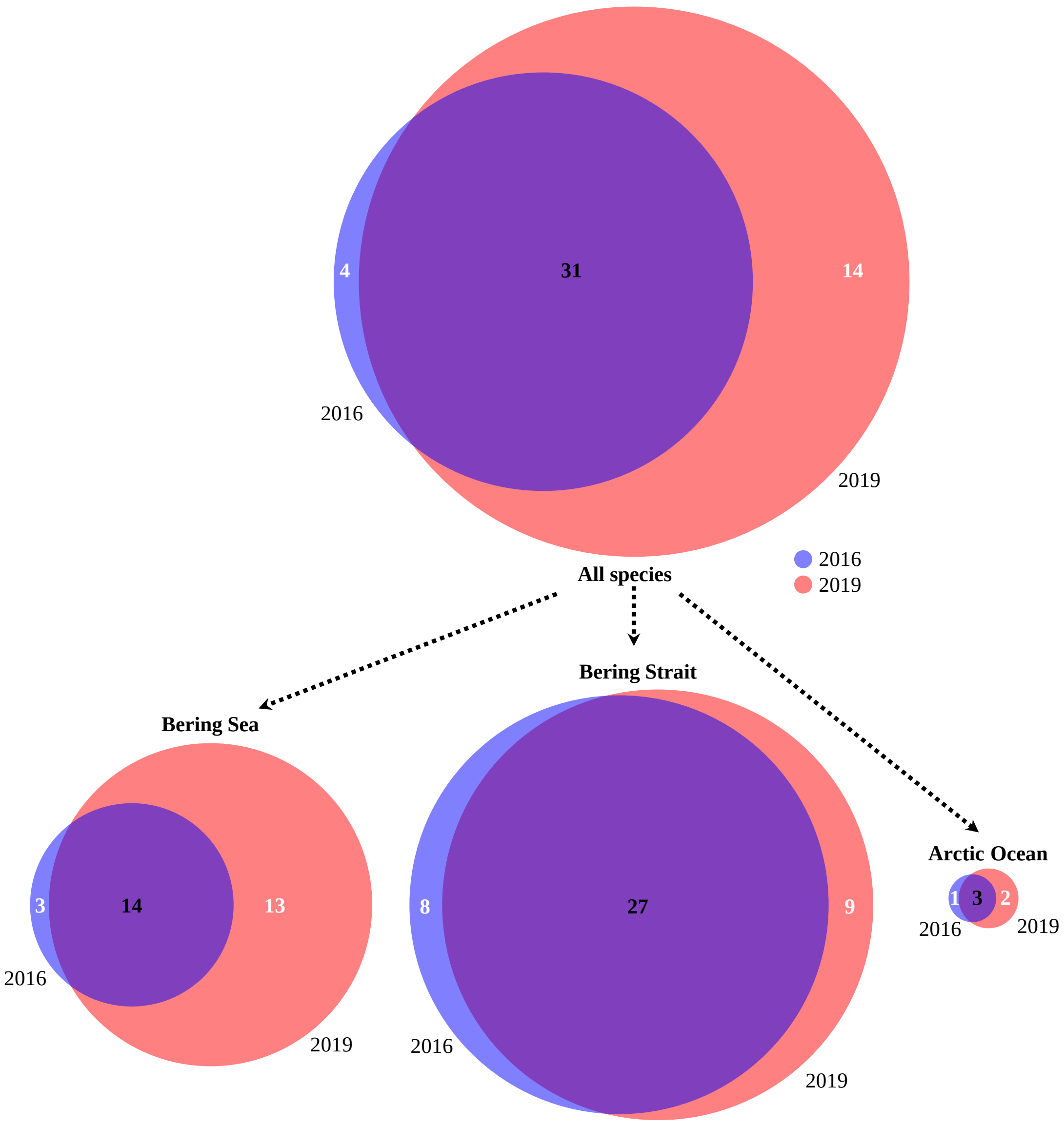

Supplementary Figure 6. Tintinnid species composition in 2016 and 2019.

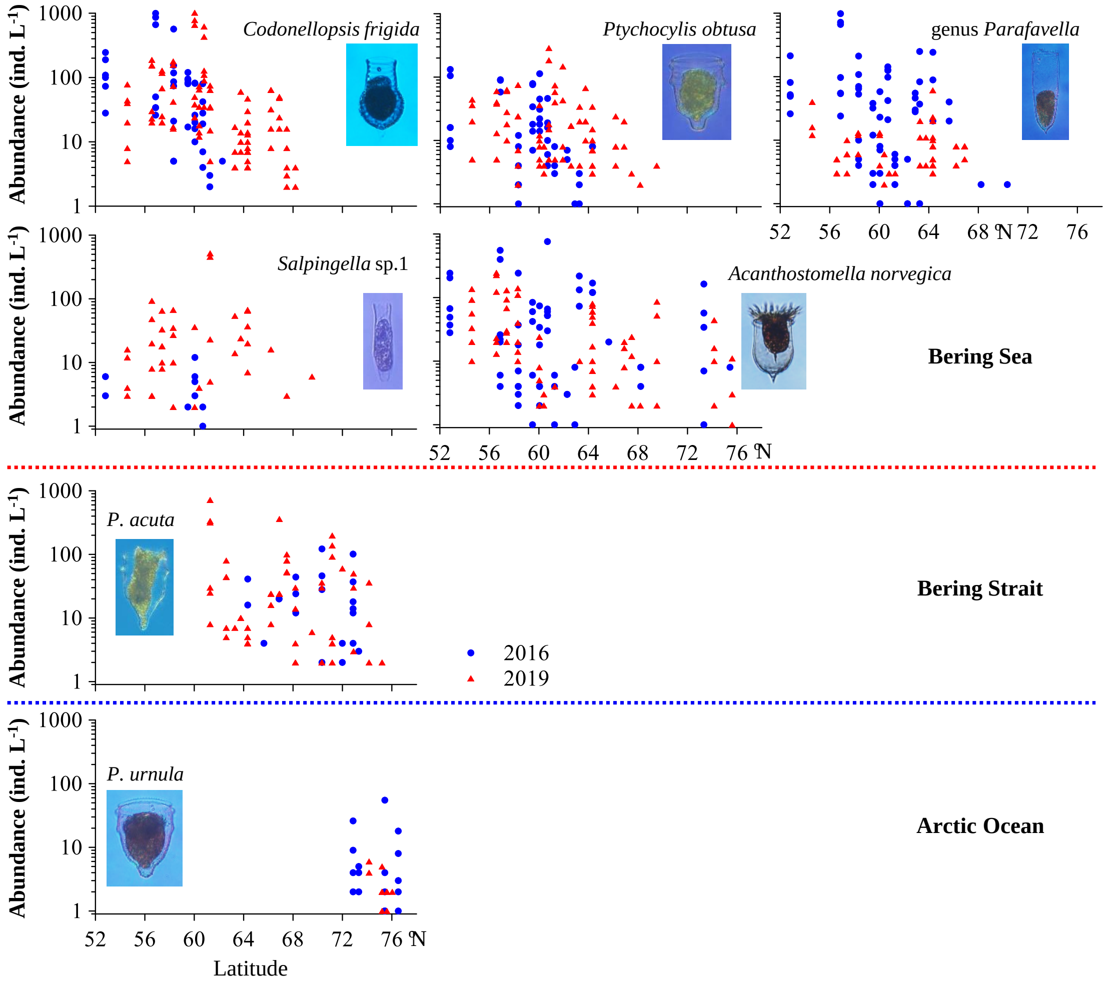

Supplementary Figure 7. Latitudinal distribution of abundant and new tintinnid species abundance in 2016 and 2019.

Supplemental material tables

Supplementary Table 1. List of stations with date sampled, environmental variables (depth (m), temperature (℃), salinity and Chl *a* concentration (Chl *a*, μg L^{-1})) and ciliate abundance range (AR, ind. L^{-1}), integrated abundance (IA, $\times 10^6$ ind. m^{-2}), biomass range (BR, μg C L^{-1}) and integrated biomass (IB, mg C m^{-2}) from surface to 200 m depth.

Station	Date sampled	Longitude	Latitude	Depth	Temperature	Salinity	Chl *a*	AR	IA	BR	IB
A1	7/18/2016	169.13 °E	52.81 °N	5917	2.9 – 10.3	32.7 – 33.9	0.0 – 0.7	273 - 1310	0.54	0.50 – 4.19	1.41
A2	7/19/2016	174.52 °E	56.87 °N	3717	1.8 – 10.1	32.9 – 33.5	0.0 – 0.5	170 - 3267	0.83	0.51 – 12.41	3.34
A3	7/20/2016	176.87 °E	58.33 °N	3670	1.8 – 10.2	33.0 – 33.6	0.0 – 0.6	152 - 1879	0.52	0.29 – 5.87	1.86
A4	9/10/2016	179.58 °W	59.48 °N	3124	2.9 – 10.5	32.8 – 33.2	0.1 – 1.4	296 - 674	0.41	0.67 – 2.11	1.13
A5	9/10/2016	179.62 °W	60.05 °N	1839	2.9 – 11.2	32.7 – 33.4	0.1 – 1.6	250 - 2592	0.66	0.56 – 5.01	1.54
A6	7/23/2016	178.51 °W	60.69 °N	3662	3.0 – 10.5	32.8 – 33.1	0.0 – 1.0	225 - 3206	0.68	0.53 – 3.99	1.34
A7	9/10/2016	177.31 °W	61.26 °N	124	1.9 – 11.1	32.5 – 32.8	0.1 – 0.6	97 - 1535	0.40	0.26 – 3.93	1.00
A8	9/9/ 2016	174.54 °W	62.28 °N	70	-1.3 – 9.6	31.0 – 31.8	0.1 – 0.6	294 - 1440	0.88	0.79 – 3.15	1.70
A9	9/9/2016	173.47 °W	62.90 °N	70	-1.4 – 8.3	31.3 – 32.4	0.2 – 0.3	386 - 1514	0.87	1.06 – 2.44	1.79
A10	9/9/2016	172.31 °W	63.26 °N	61	-1.2 – 7.5	31.7 – 32.4	0.4 – 0.7	546 - 1507	1.03	1.02 – 3.24	1.85
A11	9/8/2016	171.00 °W	64.33 °N	40	1.6 – 5.5	27.6 – 31.2	3.6 – 6.2	656 - 1356	1.10	1.32 – 4.71	3.47
A12	9/5/2016	168.71 °W	65.64 °N	50	1.9 – 8.6	30.1 – 32.6	0.7 – 2.7	956 - 1672	1.13	0.98 – 2.75	1.55
A13	9/4/ 2016	169.00 °W	66.87 °N	45	3.3 – 6.7	31.6 – 32.4	2.5 – 6.5	774 - 1194	1.09	2.0 – 4.04	3.43
A14	9/4/2016	169.00 °W	68.22 °N	57	3.4 – 8.2	31.1 – 32.1	0.8 – 2.3	245 - 419	0.34	0.31 – 1.07	0.77
A15	9/2/2016	169.00 °W	70.34 °N	39	0.8 – 6.1	31.0 – 32.3	0.3 – 1.4	994 - 2470	1.44	1.27 – 7.36	5.47
A16	9/2/2016	169.00 °W	71.99 °N	51	-0.1 – 1.6	27.0 – 32.3	0.2 – 2.8	196 - 590	0.39	0.10 – 0.49	0.32
A17	9/1/2016	169.00 °W	72.86 °N	61	-1.5 – 1.3	27.6 – 32.6	0.2 – 0.9	471 - 863	0.66	1.11 – 4.28	2.95

A18	8/24/2016	169.00 °W	73.32 °N	153	-1.8 – (-0.6)	26.7 – 32.9	0.1 – 1.0	78 - 1345	0.58	0.22 – 6.91	2.17
A19	8/23/2016	169.00 °W	75.43 °N	358	-1.3 – (-1.6)	28.0 – 33.0	0.0 – 1.5	48 - 633	0.24	0.08 – 1.76	0.56
A20	8/23/2016	169.00 °W	76.52 °N	2142	-1.2 – (-1.4)	27.0 – 32.8	0.0 – 0.4	84 - 382	0.16	0.13 – 0.96	0.37
B1	8/24/2019	171.87 °E	54.58 °N	3867	3.8-10.3	32.7-33.5	0.01-0.98	182 - 3256	0.87	0.61 – 13.43	3.04
B2	8/24/2019	174.57 °E	56.57 °N	3866	2.7-11.1	32.9-33.6	0.02-0.63	288 - 2529	0.85	0.97 – 9.26	2.89
B3	8/24/2019	175.61 °E	57.39 °N	3773	2.5-11.0	33.0-33.8	0.02-0.46	494 - 1319	0.74	1.42 – 5.39	2.88
B4	8/25/2019	177.42 °E	58.30 °N	3748	2.8-10.9	32.9-33.6	0.02-0.52	437 - 5626	1.32	1.07 – 17.24	4.76
B5	8/27/2019	179.51 °W	60.04 °N	1521	3.4-11.1	32.8-33.3	0.02-3.98	332 - 2278	0.75	1.10 – 7.55	2.62
B6	8/27/2019	179.00 °W	60.40 °N	1435	3.1-10.7	32.9-33.2	0.01-0.77	333 - 2210	0.60	0.70 – 6.83	1.96
B7	8/27/2019	178.21 °W	60.80 °N	157	2.3-11.2	32.9-33.0	0.02-2.89	561 - 4179	1.27	2.04 – 21.31	7.40
B8	8/28/2019	177.24 °W	61.29 °N	118	2.4-10.8	32.5-32.9	0.03-0.56	420 - 2227	1.18	1.21 – 14.60	7.17
B9	8/28/2019	176.18 °W	61.93 °N	113	1.5-10.2	32.3-32.6	0.06-0.91	276 - 1346	0.64	1.28 – 4.42	2.29
B10	8/28/2019	175.01 °W	62.59 °N	76	0.0-10.4	32.1-32.3	0.09-0.99	363 - 2185	1.05	1.05 – 18.23	7.42
B11	8/28/2019	173.44 °W	63.29 °N	66	0.2-10.6	32.2-32.6	0.20-0.90	857 - 2945	1.48	2.39 – 7.73	4.24
B12	8/28/2019	172.41 °W	63.77 °N	44	1.0-10.2	32.2-32.9	0.09-1.38	381 - 3250	1.63	0.76 – 7.30	4.42
B13	8/30/2019	168.75 °W	66.21 °N	55	2.4-7.6	31.7-32.8	2.87-17.75	1078 - 2413	1.54	1.74 – 6.02	3.62
B14	8/30/2019	168.75 °W	66.89 °N	43	2.6-6.3	32.4-32.7	1.80-7.61	893 - 2844	1.42	1.58 – 16.56	5.86
B15	8/30/2019	168.75 °W	67.49 °N	51	2.7-8.8	31.9-32.7	1.27-8.53	716 - 1815	1.17	3.09 – 17.92	6.61
B16	8/30/2019	168.76 °W	68.19 °N	60	3.1-6.8	32.6-32.9	0.43-2.01	406 - 1244	0.82	1.95 – 5.91	4.08
B17	8/30/2019	168.75 °W	69.53 °N	51	5.8-9.7	30.5-32.5	0.23-1.55	324 - 982	0.57	0.65 – 2.10	1.34
B18	8/31/2019	168.75 °W	70.33 °N	41	0.8-8.9	31.7-32.6	0.50-0.80	824 -5250	2.43	2.83 – 28.60	10.41
B19	8/31/2019	168.75 °W	71.17 °N	49	2.5-8.5	30.8-32.6	0.16-0.57	958 - 3548	1.84	4.36 – 27.88	13.96
B20	8/31/2019	168.74 °W	71.99 °N	50	1.9-8.0	30.8-32.8	0.75-2.04	781 - 4145	1.94	5.09 – 25.63	12.28
B21	8/31/2019	168.74 °W	72.90 °N	61	-1.5-5.1	29.3-32.6	0.18-0.94	458 – 1546	0.90	3.61 – 24.49	10.50

B22	8/31/2019	168.75 ℃W	74.16 ℃N	172	-1.5-4.8	29.0-34.4	0.02-0.47	111 - 1365	0.39	0.12 – 8.75	2.39
B23	9/02/2019	172.01 ℃W	75.21 ℃N	480	-1.6-0.9	28.0-34.6	0.01-0.22	109 - 594	0.25	0.26 – 3.00	0.82
B24	9/02/2019	172.00 ℃W	75.61 ℃N	1498	-1.6-0.5	28.2-34.5	0.01-0.28	37 - 1063	0.26	0.12 – 5.04	1.13
B25	9/02/2019	171.98 ℃W	76.03 ℃N	2012	-1.6-0.1	28.1-34.4	0.01-0.21	110 - 1009	0.36	0.16 – 3.10	1.01

Supplementary Table 2. Tintinnid species maximum abundance (A_{max}, ind. L^{-1}) and occurrence frequency (OF, %) in 2016 and 2019 from the Bering Sea to Arctic Ocean.

			**Bering Sea**				**Bering Strait**				**Arctic Ocean**			
			2016	2016	2019	2019	2016	2016	2019	2019	2016	2016	2019	2019
Type	Genera	Species	A_{max}	OF	A_{max}	OF	A_{max}	OF	A_{max}	OF	A_{max}	OF	A_{max}	OF
Cosmopolitan	*Acanthostomella*	*Acanthostomella norvegica*	553	97.0	245	83.3	752	42.4	86	28	8	8.3	11	16.7
	Amphorellopsis	*Amphorellopsis quinquealata*	1	3.0	14	27.8	5	3.0	-	-	-	-	-	-
	Amphorides	*Amphorides laackmanni*	-	-	36	11.1	-	-	-	-	-	-	-	-
	Codonellopsis	*Codonellopsis frigida*	995	100	1016	100	80	13.6	620	49.5	-	-	-	-
	Eutintinnus	*Eutintinnus pectinis*	-	-	8	2.8	12	1.5	27	11.2	-	-	-	-
		Eutintinnus sp.1	-	-	-	-	-	-	484	37.4	-	-	-	-
	Parundella	*Parundella caudata*	-	-	5	2.8	-	-	-	-	-	-	-	-
	Salpingella	*Salpingella acuminata*	68	57.6	32	52.8	14	4.5	17	2.8	7	16.7	-	-
		S. faurei	92	42.4	33	77.8	1	4.5	270	6.5	3	25	1	5.6
		Salpingella sp.1	12	21.2	93	66.7	2	3	517	15.0	-	-	-	-
		Salpingella sp.2	-	-	167	50	-	-	1405	20.6	-	-	-	-
		Salpingella sp.3	-	-	10	5.6	-	-	-	-	-	-	-	-
Boreal	*Parafavella*	*Parafavella cylindrica*	-	-	3	2.8	-	-	-	-	-	-	-	-
		P. denticulata	-	-	8	5.6	-	-	-	-	-	-	-	-
		P. elegans	27	36.4	-	-	210	25.8	4	2.8	-	-	-	-
		P. faceta	840	27.3	4	11.1	3	6.1	-	-	-	-	-	-
		P. gigantea	7	33.3	12	19.4	8	12.1	5	3.7	-	-	-	-
		P. jorgenseni	213	72.7	13	25	163	34.8	47	28	-	-	-	-
		P. promissa	-	-	-	-	1	4.5	7	2.8	-	-	-	-
		P. rotundata	10	6.1	3	2.8	3	4.5	-	-	-	-	-	-
		P. subrotundata	288	54.5	4	8.3	14	9.1	-	-	-	-	-	-

		P. ventricosa	7	9.1	8	25	2	3	14	0.9	-	-	-	-
		Total *genus Parafavella*	983	97	40	55.6	249	43.9	61	31.8	-	-	-	-
	Ptychocylis	*Ptychocylis acuta*	-	-	-	-	122	31.8	717	51.4	-	-	2	5.6
		P. obtusa	132	84.8	75	88.9	46	25.8	287	36.4	-	-	-	-
		P. urnula	-	-	-	-	26	10.6	8	7.5	55	91.7	5	44.4
Neritic	*Favella*	*Favella panamensis*	-	-	-	-	12	7.6	-	-	-	-	-	-
	Helicostomella	*Helicostomella subulata*	-	-	12	5.6	4	1.5	208	43.9	-	-	-	-
	Leprotintinnus	*Leprotintinnus pellucidus*	2	12.1	-	-	41	13.6	118	18.7	-	-	-	-
	Metacylis	*Metacylis conica*	-	-	10	5.6	-	-	-	-	-	-	-	-
	Stenosemella	*Stenosemella nivalis*	-	-	-	-	40	4.5	-	-	-	-	-	-
		S. olive	-	-	-	-	40	3	20	2.8	-	-	-	-
		S. ventricosa	-	-	-	-	43	9.1	20	6.5	-	-	-	-
	Tintinnopsis	*Tintinnopsis acuminata*	-	-	4	2.8	20	19.7	24	11.2	-	-	-	-
		T. baltica	-	-	-	-	-	-	174	38.3	-	-	-	-
		T. beroidea	-	-	-	-	36	3	-	-	-	-	-	-
		T. brasiliensis	8	3	-	-	43	31.8	110	41.1	-	-	1	5.6
		T. fimbriata	-	-	-	-	-	-	18	8.4	-	-	-	-
		T. glans	-	-	4	2.8	-	-	14	6.5	-	-	-	-
		T. karajacensis	-	-	-	-	4	4.5	20	35.5	-	-	-	-
		T. kofoidi	-	-	-	-	32	1.5	-	-	-	-	-	-
		T. levigata	-	-	-	-	-	-	10	7.5	-	-	-	-
		T. lohmanni	-	-	-	-	-	-	8	2.8	-	-	-	-
		T. parva	-	-	-	-	32	13.6	24	22.4	-	-	-	-
		T. rapa	-	-	-	-	100	47	72	53.3	-	-	-	-
		T. strigosa	-	-	-	-	4	3.0	15	12.1	-	-	-	-
		T. tubulosoides	-	-	4	2.8	8	3.0	74	40.2	-	-	-	-

	T. turbo	-	-	-	-	-	-	27	14	-	-	-	-
	T. urnula	-	-	-	-	88	7.6	107	16.8	-	-	-	-
	Tintinnopsis sp.1	-	-	3	2.8	-	-	37	11.2	-	-	-	-

Note. Species in red were abundant species with $A_{max} \geq 100$ ind. L^{-1} and OF ≥ 40%.

Supplementary Table 3. Average integrated abundance (AIA, $\times 10^6$ ind. m^{-2}) and its percentage (%) of three tintinnid genera in 2016 and 2019 in the Bering Sea, Bering Strait and Arctic Ocean.

**Seas**	**Genera**	**2016**		**2019**	
		**AIA**	**Percentage**	**AIA**	**Percentage**
**Bering Sea**	Cosmopolitan	1.87 ±0.86	66.65 ±6.46	1.40 ±0.63	87.37 ±4.23
	Boreal	1.04 ±0.85	32.99 ±6.46	0.19 ±0.11	11.82 ±5.13
	Neritic	0.01 ±0.01	0.36 ±0.61	0.01 ±0.01	0.81 ±1.13
	Total	2.92 ±1.70	100	1.60 ±0.70	100
**Bering Strait**	Cosmopolitan	0.33 ±0.55	23.20 ±29.66	0.67 ±0.79	29.65 ±27.25
	Boreal	0.39 ±0.46	28.71 ±27.92	0.45 ±0.56	31.70 ±24.33
	Neritic	0.28 ±0.47	38.09 ±36.32	0.56 ±0.48	38.65 ±20.16
	Total	1.00 ±1.02	100	1.68 ±1.39	100
**Arctic Ocean**	Cosmopolitan	0.01 ±0.00	20.56 ±7.42	0.00 ±0.01	28.86 ±35.57
	Boreal	0.07 ±0.03	79.44 ±7.42	0.01 ±0.01	69.76 ±37.88
	Neritic	0.00 ±0.00	0	0.00 ±0.00	1.38 ±2.39
	Total	0.08 ±0.03	100	0.01 ±0.01	100
